# The Current Research Landscape on the Artificial Intelligence Application in the Management of Depressive Disorders: A Bibliometric Analysis

**DOI:** 10.3390/ijerph16122150

**Published:** 2019-06-18

**Authors:** Bach Xuan Tran, Roger S. McIntyre, Carl A. Latkin, Hai Thanh Phan, Giang Thu Vu, Huong Lan Thi Nguyen, Kenneth K. Gwee, Cyrus S. H. Ho, Roger C. M. Ho

**Affiliations:** 1 Institute for Preventive Medicine and Public Health, Hanoi Medical University, Hanoi 100000, Vietnam; 2 Bloomberg School of Public Health, Johns Hopkins University, Baltimore, MD 21218, USA; carl.latkin@jhu.edu; 3 Institute of Medical Science, University of Toronto, Toronto, ON M5S 1A8, Canada; roger.mcintyre@uhn.ca; 4 Mood Disorders Psychopharmacology Unit, University Health Network, Toronto, ON M5G 2C4, Canada; 5 Department of Psychiatry, University of Toronto, Toronto, ON M5T 1R8, Canada; 6 Department of Toxicology and Pharmacology, University of Toronto, Toronto, ON M5S 1A8, Canada; 7 Institute for Global Health Innovations, Duy Tan University, Da Nang 550000, Vietnam; haipt.ighi@gmail.com (H.T.P.); huong.ighi@gmail.com (H.L.T.N.); 8 Center of Excellence in Evidence-based Medicine, Nguyen Tat Thanh University, Ho Chi Minh City 700000, Vietnam; giang.coentt@gmail.com (G.T.V.); pcmrhcm@nus.edu.sg (R.C.M.H.); 9 Department of Psychological Medicine, Yong Loo Lin School of Medicine, National University of Singapore, Singapore 119228, Singapore; e0012499@u.nus.edu; 10 Department of Psychological Medicine, National University Hospital, Singapore 119074, Singapore; cyrushosh@gmail.com; 11 Institute for Health Innovation and Technology (iHealthtech), National University of Singapore, Singapore 119077, Singapore

**Keywords:** artificial intelligence, machine learning, depression, depressive disorders, bibliometric analysis

## Abstract

Artificial intelligence (AI)-based techniques have been widely applied in depression research and treatment. Nonetheless, there is currently no systematic review or bibliometric analysis in the medical literature about the applications of AI in depression. We performed a bibliometric analysis of the current research landscape, which objectively evaluates the productivity of global researchers or institutions in this field, along with exploratory factor analysis (EFA) and latent dirichlet allocation (LDA). From 2010 onwards, the total number of papers and citations on using AI to manage depressive disorder have risen considerably. In terms of global AI research network, researchers from the United States were the major contributors to this field. Exploratory factor analysis showed that the most well-studied application of AI was the utilization of machine learning to identify clinical characteristics in depression, which accounted for more than 60% of all publications. Latent dirichlet allocation identified specific research themes, which include diagnosis accuracy, structural imaging techniques, gene testing, drug development, pattern recognition, and electroencephalography (EEG)-based diagnosis. Although the rapid development and widespread use of AI provide various benefits for both health providers and patients, interventions to enhance privacy and confidentiality issues are still limited and require further research.

## 1. Introduction

Depression is among the most common psychiatric conditions with a lifetime prevalence of 10.8% [1]. Depression is characterized by low mood, loss of interest, low energy level, poor sleep, poor appetite, suicidal thought, and poor concentration [2]. Along with anxiety, these problems may result in chronic detrimental impairments and even lead to suicidal ideation [3], medical comorbidity [4,5,6], unhealthy lifestyles [7], and unproductivity [8]. According to the World Health Organization (WHO) report, there have been over 300 million people living with depression and nearly 800,000 patients die due to suicide each year [9]. Depressive disorders caused over 50 million Years Lived with Disability (YLD) worldwide, accounting for 7.5% of global total YLD, and thus, are regarded as the largest contributor to non-fatal health loss [10].

Artificial intelligence (AI)-based techniques have been widely applied in mental health services and depression in particular. AI plays a decisive role in the fourth industrial revolution, named Industry 4.0 [11,12]. The field of AI research was first found as a research discipline “to proceed on the basis of the conjecture that every aspect of learning or any other feature of intelligence can, in principle, be so precisely described that a machine can be made to simulate it” at Dartmouth College in 1956 [13,14]. In the present age, AI can be described as the intelligence performed by computational systems, in which machines and devices are able to mimic human cognitive functions, such as learning, reasoning, and solving problem, and, thus, give rise to a broad range of applications, including robotics, machine learning, reasoning and decision making, natural language processing, and computer visions [15,16]. Due to the great potential of AI, only a few years after the establishment of this research area, physicians started to apply the reasoning foundations of intelligent techniques into a medical diagnosis procedure [17]. The field of AI has fostered the speed, accuracy, and quality of diagnosis and treatment, especially in radiology, dermatology, and pathology [18,19,20]. The last few years have witnessed a remarkable development of medical AI, especially its applications in diagnosis and treatments of depression [21,22,23], which suggests the growing interest in the field of AI-related research on depression and its interventions. Dinga et al. proposed a machine learning approach, which provides technological devices with the ability to automatically analyze and “learn” from previous data to the prediction of naturalistic courses of depression based on psychological, biological, and clinical data [22]. Machine learning was also utilized in combination with neuroimaging techniques to classify and predict likely treatment outcomes of major depressive disorders [21,23]. Furthermore, deep learning, which is a branch of machine learning, was revealed to be a powerful tool to investigate the genetic basis of mood disorders [24].

Due to the involvement of AI in the medical landscape, particularly in neuroscience and research in depression, the literature on biomedical applications of AI in this domain has rapidly expanded in recent years, which requires a comprehensive review of current research trends and patterns of this research domain. One of the few attempts to address this issue is a systematic review by Senders et al., which highlighted the great potential and effectiveness of machine learning in predicting neuropsychiatric outcomes [25]. Another systematic review conducted by Wongkoblap et al. also highlighted the trend of researching psychiatric symptoms based on the social network and other machine-learning-based techniques [26]. Nevertheless, to the best of our knowledge, there is currently no systematic review and bibliometric analysis related to the medical literature about the applications of AI in depression.

In order to demonstrate the research trends as well as identify the research gaps of AI-based research for depression, we applied bibliometric analysis, which objectively evaluates the productivity of global researchers or institutions in this field [27]. In this study:

- The trend of published articles over time and international growth based on databases of existing literature were examined.
- Current research interests on AI application in depression were pointed out.
- The development and productivity of AI research in depression was evaluated.
- The research gaps in the application of AI in depression were identified.

The organization of the paper is presented as follows. The Materials and Methods in Section 2 describes the research procedure in brief. The Results in Section 3 presents the results of this study along with the detailed interpretation. The Discussion in Section 4, as its name may suggest, further discusses the results as well as puts forward some implications. Lastly, the Conclusions in Section 5 summarize the findings of this study.

## 2. Materials and Methods

### 2.1. Search Strategy

We searched and retrieved all papers related to AI in Depression available on the Web of Science. The full search strategy was presented in the cited paper [28]. In this analysis, we selected all documents of the retrieved data on AIs that were related to “Depression” and “Anxiety.”.

### 2.2. Data Extraction, Inclusion, and Exclusion Criteria

We downloaded all data from the Web of Sciences (WoS) database in txt format, including all papers’ information such as authors’ names, papers’ title, journals’ name, keywords, institutional affiliations, frequency of citation, subject category, and abstracts. All of these data were converted to an xls. file (Microsoft Excel) to check data error. A process of standardization was carried out by two researchers to bring together the different names of an author. We filtered all downloaded data by excluding the papers, which were (i) not original articles and reviews, (ii) not about Depression and AIs, and (iii) not in English. Any articles that may not have been aligned with the objectives were thoroughly discussed. The combined dataset was transferred into Stata for further analysis.

### 2.3. Data Analysis, Outcomes, and Data Synthesis

Data were analyzed based on basic characteristics of publication (number of authors, publication years, main category), keywords (most common keywords and co-occurrence keywords), citations, usages, and abstracts. After downloading and extracting the data, we applied descriptive statistical analysis using Stata to calculate country citations and inter-country collaboration. A network graph illustrating the connection among countries by sharing the co-authorships was created, along with the author keywords’ co-occurrence network. VOSviewer software (version 1.6.8, Center for Science and Technology, Leiden University, the Netherlands) was used to extract the authors’ keywords and establish a co-occurrence network of the most common ones. For further content analysis of the abstracts, we applied exploratory factor analysis, which is a statistical method uncovering the underlying association between a variable set, to identify co-occurring terms and, thus, uncover the major research domains from all content of the abstracts [29]. Latent Dirichlet Allocation (LDA), which is a generative probabilistic model for collections of discrete data including text corpora, was used for classifying papers into corresponding topics [30,31,32,33,34]. The summary of analytical techniques for each data type is presented in Table 1.

## 3. Results

### 3.1. Systematic Search Results

The very first paper on the use of AI in depression was published in 1993, which was followed by six consecutive years without any publication. The last decade had witnessed the significant rise of interest in the applications of AI in depression studies and interventions. From 2010, the total number of papers had risen significantly and reached 117 publications in 2018, which doubled that of the previous year and accounted for nearly 30% of total articles of all years. Besides the dramatic increase in the number of papers, the total citations of 2018 were substantial, even though the articles were published in one year only. Since the rise of AI could assist physicians and psychiatrists in shortening processing times and improving the quality of care in clinical practice, it is no surprise that the potential of AI in medicine has attracted more attention from researchers, which makes the year 2018 an important year for AI research [11,35]. The total usage and mean use rate of AI in depression research had increased considerably, especially in the last five years (Table 2).

The number of publications counted by study settings of 25 countries is presented in Table 3. The bibliography included country settings were 79 times in total, and, of those, the US attributed 26 times, accounting for 32.9%. Over 90% of the total settings were performed in developed countries, likely due to the technological advancements of these nations. 

### 3.2. Global Network of AI Research in Depression

**Figure 1:**
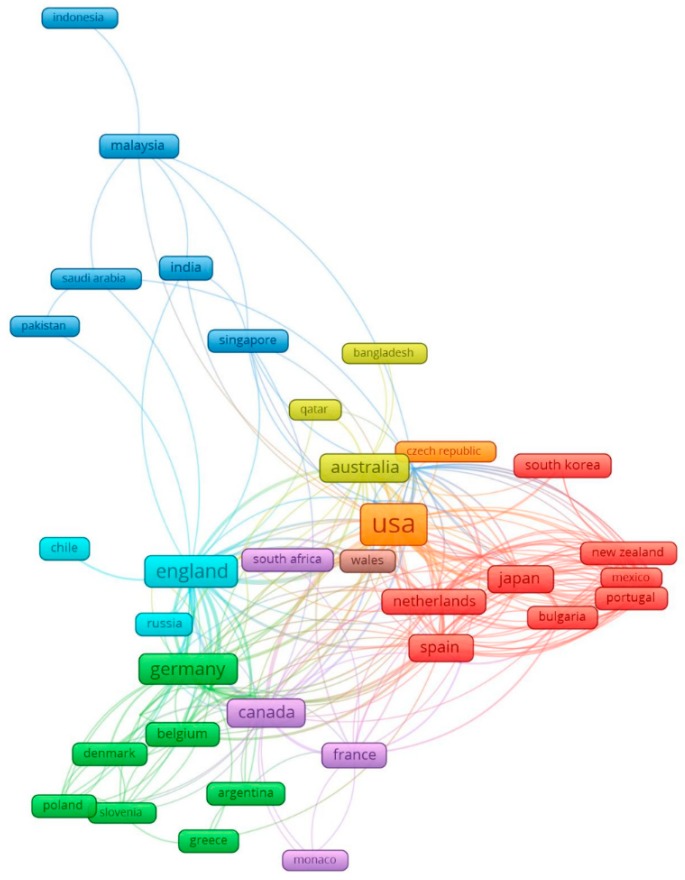
The global networking of 55 countries having at least five co-authorships are classified in six clusters.

Figure 1 presents the global network among 55 countries collaborating among selected publications. The US ranked first at the number of publication and collaboration networks [36]. Countries such as the United Kingdom (UK), Germany, France, Belgium, and Denmark, which possess the most advanced digital technologies in the Europa [37], had strongly collaborated to promote research in AI for managing depression. The red cluster reveals that Japan and South Korea have also joined this research field by collaborating with highly developed Western AI researchers (Netherlands, Spain, and New Zealand) and proved their potentials to be important researchers in this field [38,39]. In the meanwhile, India and South-East Asian countries, including Singapore, Malaysia, and Indonesia (blue nodes), were highly connected, possibly due to the similar measures of trade and financial flows [40].

### 3.3. Key Research Subtopics in AI Research for Depression

**Figure 2:**
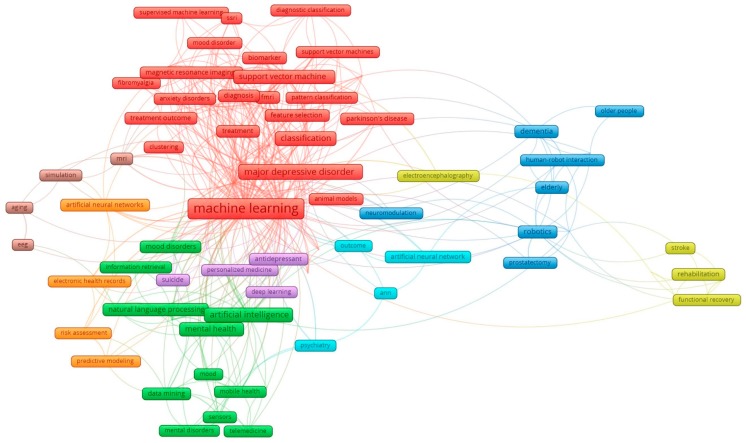
Co-occurrence of most frequent research keywords. Note: the colors of the nodes indicate principle components of data structure. The nodes’ size was scaled to the keywords’ occurrences. The thickness of the lines was drawn based on the strength of the association between two keywords.

Analyses of keywords and the abstract content provide us with a better understanding of the scopes of studies and development of research landscapes. The principal components of keywords’ structure with the most frequent groups of terms are displayed in Figure 2. The clusters were merged from 85 most frequent keywords with a co-occurrence of three times and higher. Nodes in red refer to machine learning and such applications as diagnostic classification, functional magnetic resonance imaging (fMRI), and support vector machines, in treatment and diagnosis for various neuropsychiatric, including Parkinson’s disease, fibromyalgia, mood disorder, major depressive disorder, and anxiety disorders. Green cluster describes how AI is applied in interventions for mental health, while the blue cluster focuses on the use of robots in assisting the elderly with dementia. Meanwhile, the other nodes mention a number of neuropsychiatric diagnostic tests and techniques.

As for the content analysis of abstracts by exploratory factor analysis, the top 50 emerging research domains are listed in Table 4. The most well-studied application of AI in depression appeared to be the utilization of machine learning to predict clinical characteristics, which accounted for more than 60% of total cases. Researchers have also paid attention to a wide range of issues and fields where AI can foster innovation, such as diagnosis accuracy, structural imaging techniques, negative symptoms of certain disorders and diseases, gene testing, and drug development.

### 3.4. Exploratory Factor Analysis

**Figure 3:**
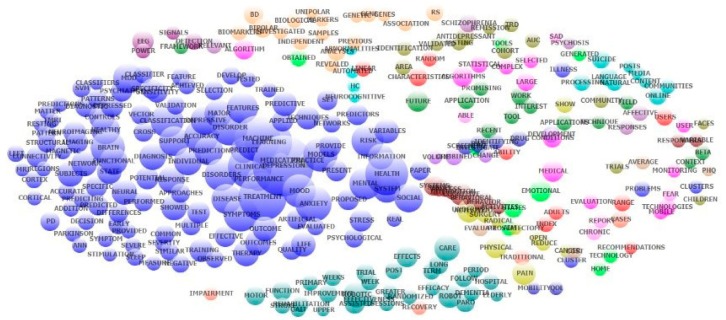
Co-occurrence of the most frequent topics emerged from exploratory factor analysis of abstract content.

Exploratory factor analysis of abstracts’ contents showed the most frequent research topics (Figure 3). A wide range of research domains, such as symptoms of depression, anxiety, and depression in Parkinson’s disease and other neuropsychiatric disorders, machine learning, and predictive models, diagnostic techniques (neuroimaging, MRI, fMRI) and their accuracy, and treatment outcomes, are specified in cobalt blue cluster. Steel blue nodes, which are located at the bottom of the figure, focus on both short-term and long-term randomized trials targeting the elderly to evaluate the effects of using robots in hospital healthcare services and rehabilitation to reduce depression. Nodes in orange focus on biological analyses through genes and biomarkers, whereas the remaining scatters on the right of the figure mainly refer to technological issues. Statistical characteristics, development of mobile technologies, natural language processing, promising applications, and future works, for instance, are the main research topics.

In Table 5, we present the research topics identified by the Latent Dirichlet Allocation. The labels of the topics were annotated by scrutinizing the most frequent words and titles for each topic. Topics with the largest volumes of publications included (i) pattern recognition, neuro-morphometrics, and neuroimaging with 17.9% of total papers focusing on this topic, and (ii) electroencephalography (EEG)-based diagnosis (17.1%), which were by far higher than other research areas.

**Figure 4:**
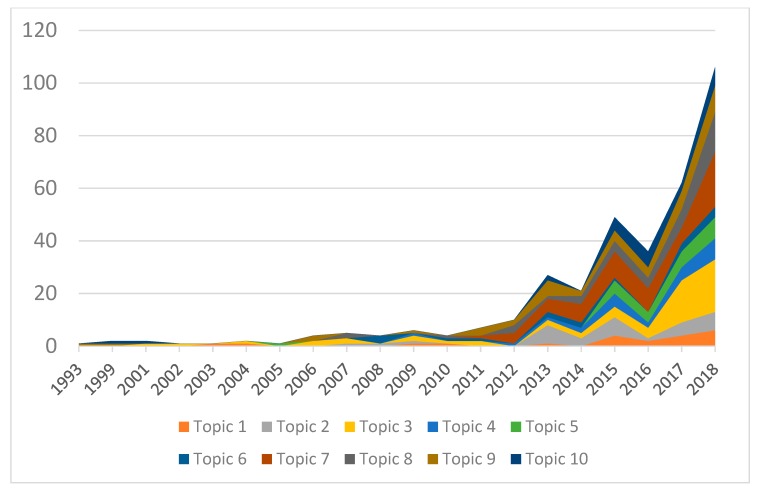
Changes in applications of AI to depression research during 1991–2018.

The changes in research productivity over time is illustrated in Figure 4. Significantly more publications were produced throughout the research period, which indicates that the applications of AI in depression attracted more attention, especially during the last few years, as illustrated by a sharp increase from 2016 to 2018. There is no surprise that pattern recognition, neuro-morphometrics, and neuroimaging (topic 7) comprised the greatest percentage of total volume (Table 4), since AI developers and researchers had shown strong interest in this area for nearly a decade. Meanwhile, the EEG and AI-assisted diagnosis (topic 3) had been extensively studied, which indicates the relatively rapid growth of attention on the applications of EEG and AI in assessing depression.

**Figure 5:**
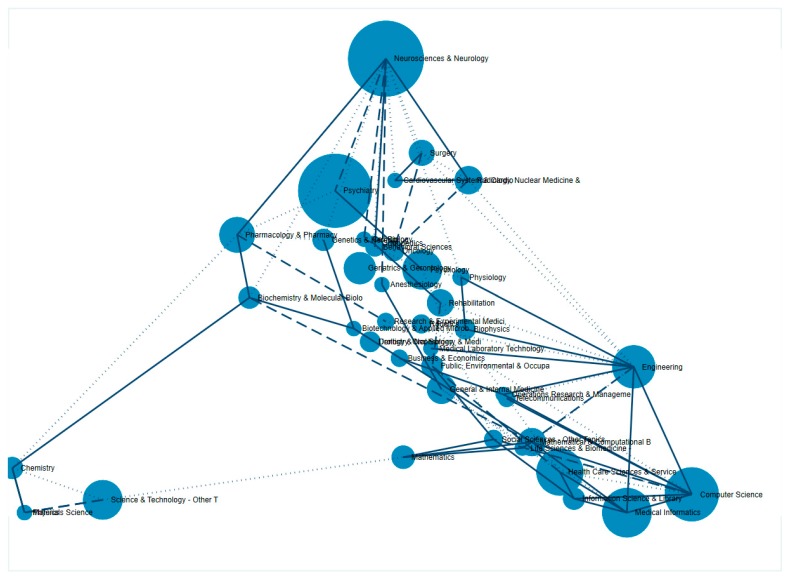
Coincidence of research areas using the Web of Science classifications.

Figure 5 shows the coordinates of principles research areas based on the WoS classification. Using principle components analysis, the research areas have been formed into five multidisciplinary categories, including (i) neuroscience and psychiatry, (ii) biomedical sciences, (iii) pharmacology, (iv) mathematics and informatics, and (v) social sciences. Notably, computer science and biotechnology were involved in numerous fields.

## 4. Discussion

This study demonstrated the significant increase in quantity as well as trajectory in citations and usages of peer-reviewed literature that broadly evaluate AI across disparate aspects of depression. Research landscapes related to this field include clinical predictive analytics, neuropsychiatric diseases’ treatment and healthcare, and biomedical applications. Although the number of publications on applications of AI in depression studies has rapidly increased in recent years, to the best of our knowledge, this study can be considered the first one providing an organizational framework of the AI applications in depression based on the existing literature. Therefore, this study offers the opportunity to prioritize settings and refine further efforts.

In this day and age, when digitization provides human life with convenience and enormous benefits and possible hazards, information technology and its potential applications have attracted a great deal of attention. The last five years have witnessed the exponential growth of the interest in applications of AI in depression studies, as illustrated by the significant expansion of scientific literature on this topic (Table 1). Developed countries, such as the US, England, Germany, and Japan, have made numerous attempts to innovate the current AI system and conduct various research studies to confirm its effectiveness [37,38]. The foregoing countries have also supported and collaborated with many nations, despite the disproportionate data from the selected countries (Figure 2).

Analyses of keywords and abstract content pointed out that machine learning is among the cutting-edge and most widespread branch of AI. Machine learning provides technological devices with the ability to automatically analyze and “learn” from previous data and, thus, make accurate decisions on new datasets without explicit algorithms [41]. This subfield has been applied in diagnosing a wide range of mental disorders, such as predicting treatment outcome in depression [42], classifying dyslexia structural neuro-imaging [43], and identifying incipient dementia [44]. Algorithms of machine learning offer dynamic conceptual and analytic frameworks to integrate multiple data types and sources to predict therapeutic outcomes of depression [45]. Additionally, various biomedical techniques and applications were found to be involved in multiple research fields and were strongly associated with psychiatry as well as computer sciences and informatics. This incorporation suggests the development of a multidisciplinary AI approach for further research and findings in neuropsychiatric disorders and treatments (Figure 5).

Nevertheless, the terms “privacy” and “confidentiality” were absent from the results of keywords and abstract content analyses, which indicates a lack of attention on these particular issues. Aspects related to privacy and confidentiality are significant concerns since they relates to general health and mental health insofar as individuals with depression expect that their diagnosis and other clinical information will be private, either by government regulations or the use of technology that secure their personal data and prevent them from unauthorized usage [46]. Nonetheless, in this era of big data, since data has become the endless source of economic and social value, the accumulation and analyses of data continue to increase rapidly [47,48]. Along with the electronic storage of these data in perpetuity [49] and the joining of data sets, these processes increase the risk of privacy violation. Furthermore, despite the fact that patients’ information is usually anonymized, data re-identification techniques still pose potential threats to personal information [50] since the breach of clinical data may facilitate AI crimes [51] or other improper uses [52]. Insurance companies, for instance, may take advantage of AI to project whether their clients have severe illnesses and deny taking out the policy. In addition, there was a lack of research on treatment guidelines developed by AI and compared with treatment guidelines developed by expert psychiatrists [53].

Our findings have several implications. Developing countries, especially those from the south-east Asian region, could seek investment from developed countries in the field of information technology. The U.S., for instance, invested 23 billion USD in AI [38] in 2016, while the UK has planned to allocate €8.5 billion for commercialization of AI [37]. Other applications of AI, including artificial neural network, genetic algorithms, and natural language processing, deserve more attention and better development. Future policy should address the privacy related to the usage of big data or the collection of real-time clinical data gathered from smartphone applications among people with psychiatric conditions [54,55,56,57,58]. Since patients suffering from depression are particularly vulnerable, the leakage of clinical information may worsen their health status and possibly lead to emotional trauma. Therefore, new policy and research on the use of AI in management of depression should be made to achieve a balance between the beneficial uses of clinical data and personal privacy.

On the other hand, despite the advancements as well as achievements of medical application of AI, the major challenge is the integration and application of heterogeneous data. Since clinical data is becoming more supplicated, researchers have to handle numerous types, provenances, and massive amounts of data, such as demographic data, biomedical patterns, images, pathological data, genetic data, social network data, and different categories of data [59]. The diversity of clinical data increases the complexity and difficulty when designing algorithms and establishes reasoning models for specific clinical tasks [60]. Additionally, in some parts of the world, computers have arrived at the conclusion that it would be risky to implement policy on insurance coverage, which may deprive people of their healthcare benefits. Therefore, future AI developers, as well as policymakers, need to overcome the previously mentioned obstacles in order to further innovate and apply AI in the management of depression.

Although this bibliometric analysis contains a large body of literature based on the topic of interest with an intensive summary of keywords and research patterns, some limitations need to be considered. Since this study only included English papers, there might be a selection bias toward studies conducted in English-speaking countries. In addition, the publication type was restricted to a peer-reviewed research publication, which possibly influences the thoroughness of the analyzed results. 

Over the next three to five years, the research objectives will involve the integration of various clinical data, neuroimaging, and biochemical parameters and genotypes into the AI system, which helps diagnose depression and predict treatment outcomes. With further research and more data, the future AI system will be able to advise psychiatrists on the best choices of antidepressants and dosages, stimulation treatment (e.g., electroconvulsive therapy), or psychological therapies.

## 5. Conclusions

In conclusion, the applications of AI in the healthcare system for managing depression have become more significant. The rapid development and widespread use of AI not only provide both health providers and patients with various benefits, but the research on interventions to enhance privacy and confidentiality issues are still limited. This is one of the key challenges that governments and organizations need to safeguard in order to optimize the use of AI in mental health. Additionally, the field should move from big data analysis to clinical application, since this would provide a more personalized and evidenced-based treatment for individuals with depression. Comparing the outcome of treatment guidelines for depression developed by AI versus the outcome for depression treatment guidelines developed by expert psychiatrists would also be required in the near future.

## Figures and Tables

**Table 1:** Analytical techniques and presentations of results of each data type.

Type of Data	Unit of Analysis	Analytical Methods	Presentations of Results
Keywords, countries	Words	Frequency of co-occurrence	Map of keywords clusters
Abstracts	Words	Exploratory factors analyses	Top 50 constructed research domains. Clustering map of the landscapes constructed by these domains.
Abstracts	Papers	Latent Dirichlet Allocation	10 classifications of research topics
WoS classification of research areas	WoS research areas	Frequency of co-occurrence	Dendrogram of research disciplines

**Table 2:** General characteristics of publications.

Year Published	Total Number of Papers	Total Citations	Mean Cite Rate Per Year ^1^	Total Usage ^2^ Last 6 Month	Total Usage ^2^ Last 5 Years	Mean Use Rate Last 6 Month ^3^	Mean Use Rate Last 5 Year ^4^
2018	117	126		738	1121	6.31	1.92
2017	68	296	4.35	210	2076	3.09	6.11
2016	43	476	5.53	117	1019	2.72	4.74
2015	55	934	5.66	88	984	1.60	3.58
2014	27	577	5.34	33	478	1.22	3.54
2013	29	676	4.66	39	743	1.34	5.12
2012	12	871	12.10	41	522	3.42	8.70
2011	10	403	5.76	15	227	1.50	4.54
2010	6	159	3.31	1	40	0.17	1.33
2009	6	91	1.69	4	42	0.67	1.40
2008	4	30	0.75	0	16	0.00	0.80
2007	5	98	1.78	3	39	0.60	1.56
2006	4	10	0.21	0	5	0.00	0.25
2005	1	1	0.08	0	0	0.00	0.00
2004	2	44	1.57	0	7	0.00	0.70
2003	1	31	2.07	0	4	0.00	0.80
2002	2	31	0.97	0	4	0.00	0.40
2001	2	25	0.74	0	9	0.00	0.90
1999	2	20	0.53	0	6	0.00	0.60
1993	1	9	0.36	0	0	0.00	0.00

^1^ mean cite rate per year = total citations/(total citations × (2018-that year)). ^2^ total usage = total downloads. ^3^ mean use rates last 6 months = total usage last 6 months/total number of papers. ^4^ mean use rates last 5 years = total usage last 5 years/total number of papers × 5.

**Table 3:** Number of papers by countries as study settings.

	Country Settings	Frequency	%
1	United States	26	32.9%
2	Ireland	10	12.7%
3	United Kingdom	8	10.1%
4	Australia	3	3.8%
5	India	3	3.8%
6	New Zealand	3	3.8%
7	Spain	3	3.8%
8	China	2	2.5%
9	France	2	2.5%
10	Japan	2	2.5%
11	Netherlands	2	2.5%
12	Taiwan	2	2.5%
13	Afghanistan	1	1.3%
14	Chile	1	1.3%
15	Germany	1	1.3%
16	Hong Kong	1	1.3%
17	Iran	1	1.3%
18	Italy	1	1.3%
19	Malaysia	1	1.3%
20	Mexico	1	1.3%
21	Portugal	1	1.3%
22	South Africa	1	1.3%
23	Sweden	1	1.3%
24	Switzerland	1	1.3%
25	Wallis and Futuna	1	1.3%

**Table 4:** Top 50 research domains emerged from exploratory factor analysis of all abstracts’ contents.

Number	Name	Keywords	Eigenvalue	Frequency	% Cases
1	Predictors; Predicted	predictors; predicted; prediction; predict; predicting; clinical; characteristics; predictive; variables	1.56	568	63.0%
2	Machine Learning	learning; machine; algorithms; techniques	1.99	511	61.2%
3	Resting-State; Functional Connectivity	resting; state; connectivity; functional magnetic resonance imaging (fMRI); controls; power; classifiers; functional; linear; healthy	2.40	408	50.4%
4	Mental Health	mental; health; stress; real; problems; psychological	2.12	333	50.1%
5	Depressive	depressive; major; major depressive disorder (MDD); disorder; remission	2.69	352	45.8%
6	Diagnosis; Accuracy	diagnosis; accuracy; predictions; accurate	1.72	247	43.3%
7	Antidepressant; Treatment Response	antidepressant; response; treatment-resistant depression (TRD); treatment; remission	2.90	241	39.3%
8	Bipolar; Mood Disorders	bipolar; mood; bipolar disorder (BD); disorders	3.12	229	38.3%
9	Imaging; Structural	imaging; structural; magnetic; magnetic resonance imaging (MRI); matter; brain; functional; neuroimaging; volume	16.70	366	37.3%
10	Stroke; Rehabilitation	stroke; rehabilitation; robotic; assisted; upper; motor; function; gait; therapy; effectiveness; sessions; stimulation	5.43	308	36.8%
11	Feature Selection; Features	feature; selection; features; framework; validation	2.40	233	34.0%
12	Fear; Report	fear; report; anxiety; sad	1.67	163	33.8%
13	Field; Application	field; applications; application; future; recent	1.82	201	33.5%
14	Pain; Quality of Life (Qol)	pain; qol; follow; outcome; hospital; surgery; week; robotic; mobility	1.99	240	33.5%
15	Artificial Neural	neural; artificial; artificial neural networks (ANN); network; networks	2.66	232	31.5%
16	Trial; Randomized	trial; randomized; week; trials; outcomes; efficacy; weeks	2.57	232	31.2%
17	Faces; Fmri	faces; fmri; pattern; independent; depressed; sad; samples	2.03	181	30.2%
18	Human-Computer; Abnormalities	human-computer; abnormalities; neurocognitive; controls; healthy; automated	2.07	181	28.5%
19	Support Vector	vector; support; support vector machine (SVM); classifier	3.39	200	27.5%
20	Parkinson	parkinson; Parkinson’s disease (PD); disease; motor	2.21	146	27.0%
21	Investigated; Previous	investigated; previous; risk	1.96	133	27.0%
22	Effective; Cost	effective; cost; provided; psychological	1.91	137	26.2%
23	Paro; Dementia	paro; dementia; elderly; robot; care; sessions	2.98	148	24.7%
24	Classifiers; Process	classifiers; process; applied	1.65	118	23.9%
25	Negative Symptoms	symptoms; negative	2.19	105	22.7%
26	Behavior; Systems	behavior; systems; mobile; monitoring; technologies	2.26	127	22.2%
27	Biomarkers; Markers	biomarkers; markers; neuroimaging; patterns	2.05	122	21.9%
28	Statistical; Complex	statistical; complex; index	1.77	100	20.4%
29	Posts; Social Media	posts; media; communities; content; online; social	3.77	134	19.9%
30	Schizophrenia; Psychiatric	schizophrenia; psychiatric; illness	1.76	98	19.7%
31	Quality	quality; life; qol; mobility	2.48	128	19.7%
32	Physical Activity	activity; physical	1.69	94	19.7%
33	Cognitive Impairment	impairment; greater; cognitive	1.72	99	19.7%
34	Radical Prostatectomy; Surgery	radical; prostatectomy; surgery; cancer; underwent; open; assisted	3.35	116	18.1%
35	Area Under Curve; Area	auc; area; achieved	1.81	90	17.9%
36	Investigate	investigate; aim	1.63	78	17.1%
37	Technology; Home	technology; home; technologies; reduce	1.71	87	16.4%
38	Natural Language	language; natural; processing; suicide	2.35	111	16.1%
39	Testing	testing; identification; cohort	1.97	75	15.4%
40	Gene	genes; gene; genetic; refSNP (rs); association; interaction	4.06	96	15.1%
41	Drug	drug; development	1.62	63	14.1%
42	Sensitivity	sensitivity; specificity; suicide	2.27	101	13.9%
43	Medical	medical	1.61	48	12.1%
44	Single	single	1.55	45	11.3%
45	Detection; EEG Signals	detection; signals; electroencephalography (EEG)	1.68	58	11.1%
46	Responses; Psychosis	responses; psychosis; affective	1.91	53	10.8%
47	Beta; Adults	beta; adults; context	1.86	50	10.8%
48	Addition	addition	1.64	37	9.3%
49	Ability; Responders	ability; responders	1.58	39	9.1%
50	Primary	primary	1.53	36	9.1%

**Table 5:** Ten research topics classified by Latent Dirichlet Allocation.

Number	Research Areas	Frequency	Percent
1	Genomics and computational modeling in depression	21	6.0%
2	Depression as an outcome in AI and robotic assisted surgery	33	9.4%
3	The use of AI and electroencephalography in the diagnosis of depression	60	17.1%
4	The impact of social media and online communities on depression	25	7.1%
5	The use of AI in the psychological intervention for depression	24	6.8%
6	The use of AI to assess the use of alternative treatment	17	4.8%
7	The use of pattern recognition by artificial intelligence, neuro-morphometric, and neuro-imaging in the diagnosis of depression	63	17.9%
8	The use of biomarkers and machine learning in clinical risk stratification of depression	40	11.4%
9	Behavioral pattern monitoring and possible interventions for depression through telehealth and mobile applications	43	12.3%
10	The use of AI in electronic health records to predict the outcome of depression and suicide	25	7.1%
	Total	351	100%
